# Quality and Authenticity of Metformin Tablets Circulating on Japanese Websites

**DOI:** 10.1007/s43441-021-00262-3

**Published:** 2021-02-17

**Authors:** Shu Zhu, Naoko Yoshida, Hirohito Tsuboi, Ryo Matsushita, Kazuko Kimura

**Affiliations:** 1 Graduate School of Medical Sciences, Medi-Quality Security Institute, Kanazawa University, Kakuma-machi, Kanazawa, 920-1192 Japan; 2 AI Hospital/Macro Signal Dynamics Research and Development Center, Institute of Medical, Pharmaceutical and Health Sciences, Kanazawa University, Kakuma-machi, Kanazawa, Ishikawa 920-1192 Japan; 3 Clinical Pharmacy and Healthcare Sciences, Faculty of Pharmacy, Institute of Medical, Pharmaceutical and Health Sciences, Kanazawa University, Kakuma-machi, Kanazawa, 920-1192 Japan

**Keywords:** Poor-quality medicine, Metformin, Quality, Personal import, Internet

## Abstract

**Background:**

Low-quality medicines and falsified medicines represent long-standing problems in developing countries. In Southeast Asia, the circulation of low-quality diabetes drugs (metformin) has been confirmed. It is possible that low-quality metformin has entered Japan via personal import through the Internet. This study evaluated the pharmaceutical quality and authenticity of metformin tablets obtained via the Internet in Japan.

**Methods:**

In total, 33 samples of 500-mg metformin tablets and 7 samples of extended-release/sustained-release tablets (500, 750, and 1,000 mg) were purchased via personal import in January 2017. Confirmation of a prescription was never requested purchase. The obtained samples were subjected to visual observations and authenticity investigations. Additionally, quantitative analysis, content uniformity and dissolution tests were performed using HPLC–PDA.

**Results:**

Our authenticity investigations revealed that seven samples were genuine products, whereas the authenticity of the remaining 33 samples was unclear. Referring to United States Pharmacopeia 2014 for validation, four samples failed quality testing, five samples failed content uniformity testing, and two samples failed dissolution testing.

**Conclusions:**

Our findings illustrate that metformin tablets of poor-quantity and unregistered/unlicensed doses are available online and that it is important to increase consumer awareness about the presence of these medicines on the Internet to prevent the purchase of substandard medicines.

## Introduction

Diabetes mellitus, a metabolic disorder characterized by hyperglycemia, seriously affects human health and quality of life [1]. The World Health Organization (WHO) estimated that 108 million people had diabetes in 1980, and this number is projected to increase to 693 million by 2045 [2, 3]. Preventing and managing diabetes have become major public health challenges worldwide.

Metformin (1,1-metformin) is a biguanide derivative and is the most commonly used treatment for type 2 diabetes (T2D) [4]. Various studies have illustrated that metformin has roles in preventing diabetes, protecting the nervous and cardiovascular systems, and treating polycystic sputum syndrome and cancer in addition to T2D [4–7]. In the future development of medical treatment, metformin will play a particularly important role.

However, even if efficacious medicines are developed, patients may not benefit in some cases because of the existence of substandard and falsified medicines. For example, the active components of substandard medicines do not reach the therapeutic level, and falsified medicines may not contain therapeutic components. WHO defines “substandard and falsified medical products” as follows: “a) substandard (also called “out of specification”): these are authorized medical products that fail to meet either their quality standards or specifications, or both; b) unregistered/unlicensed: medical products that have not undergone evaluation and/or approval by the National or Regional Regulatory Authority for the market in which they are marketed/distributed or used, subject to permitted conditions under national or regional regulation and legislation; c) falsified: medical products that deliberately/fraudulently misrepresent their identity, composition, or source” [8]. It was stated in many reports that falsified and substandard medicines are available in developing countries [9–12]. However, falsified and substandard medicines appear to be available globally [13]. People in the United States, Europe, and Australia are generally aware that online pharmacies form part of the official medicine distribution channels, and controlled medicines require a prescription [14]. However, with the increasing consumption of prescription drugs, unlicensed, substandard, and falsified medicines with dubious medical claims are advertised and sold illegally by many rogue online pharmacies [15]. It is illegal to sell prescription medicines online in Japan. However, the personal import of prescription medicines is not illegal in the country [16]. Although the government does not encourage citizens to import drugs, it cannot prohibit this activity. In a research report from 2016, no more than 1% of agencies advertising medicine for personal import were in compliance with Japanese law [17]. The supply chain of pharmaceuticals is managed properly in Japan, but the personal import of medicines via the Internet might allow substandard and falsified medicines to enter the country. In previous studies, we found that falsified weight-loss and erectile dysfunction drugs containing no active pharmaceutical ingredient [18–20]. Even when drugs are not falsified, the quality of a drug produced by the same manufacturer can differ according to the place of origin and market [21] In Southeast Asia, the circulation of low-quality metformin has been confirmed [12]. However, the quality and authenticity of metformin circulating on the Internet remain unclear.

To prevent the use of substandard and falsified medicines, this research examined the quality and authenticity of metformin tablets available online.

## Materials and Methods

### Sample Collection

We used Google Japan to identify purchase websites using the keyword search “Metformin personal import” in Japanese (メトホルミン 個人輸入). Among the purchase websites that could be identified using Google Japan, all sites that permitted payment via bank transfer were targeted. We performed Internet searches between October 27, 2016 and November 9, 2016.

Based on the website search, 500-mg metformin tablets were sold on most websites. Therefore, 500-mg metformin tablets were purchased in this study. Additionally, we purchased sustained-released/extended-release tablets containing 500, 750, or 1,000 mg of metformin. The number of units purchased per site exceeded 60 tablets. Additionally, when different packaging types (boxes and bottles) were available on the same site, both were purchased (Table 1).

**Table 1:** List of samples collected

Sample No	Site No	Stated product name	Dose (mg)/number of pills	Packaging and language	Labeled manufacturing and/or distributing country	Shipping country	Package insert and language	Authenticity
1	1	GLYCIPHAGE	500/60	PTP/English	India	Singapore	None	Unknown
2	2	ZOMET	500/100	Box/English	India	Singapore	None	Unknown
3	3	GLYCOMET	500/100	PTP/English	India	Singapore	None	Unknown
4	4	GLUCOPHAGE	500/100	APCFP/English	Unknown	Thailand	None	Unknown
5		Metformin	500/56	Box/English	United Kingdom	Singapore	Yes/English	Unknown
6		APO-Metformin 500	500/100	Box/English	Unknown	Singapore	None	Genuine
7	5	APO-Metformin 500	500/100	Box/English	United Kingdom	Singapore	None	Genuine
8	6	APO-Metformin 500	500/100	Box/English	Unknown	Singapore	None	Genuine
9	7	Metformin	500/100	Bottle/English	India	America	Yes/English	Unknown
10	8	Metformin	500/56	Box/English	United Kingdom	Singapore	Yes/English	Unknown
11	9	Metformin‐GPO	500/100	APCFP/English	Thailand	Thailand	Yes/English	Unknown
12		Siamformet	500/100	Box/English and Thai	Thailand	Thailand	Yes/Thai	Unknown
13	10	Metformin	500/112	Box/English	United Kingdom	Singapore	Yes/English	Unknown
14		APO-Metformin 500	500/100	Box/English	United Kingdom	Singapore	None	Genuine
15		ZOMET	500/100	Box/English	India	Singapore	None	Unknown
16	11	ZOMET	500/100	Box/English	India	Singapore	None	Unknown
17	12	GLYCIPHAGE	500/60	PTP/English	India	Singapore/Hong Kong	None	Unknown
18	13	Glucophage	500/200	PTP/Chinese	France	Taiwan	Yes/Chinese	Unknown
19	14	Okamet-500	500/200	PTP/English	India	India	None	Genuine
20	15	GLYCOMET	500/100	Box/English	India	Singapore	None	Unknown
21	16	METCHEK	500/1000	Bottle/English	New Zealand	Hong Kong	None	Unknown
22		GLYCIPHAGE	500/60	PTP/English	India	Hong Kong	None	Unknown
23	17	Glucophage	500/280	PTP/English	Unknown	Malaysia	None	Unknown
24		Metformin HCl BP 500	500/250	PTP/English	Unknown	Malaysia	None	Unknown
25	18	GLUCOPHAGE	500/60	APCFP/English	Unknown	Thailand	None	Unknown
26	19	APO-Metformin 500	500/100	Box/English	Unknown	Singapore	None	Genuine
27		Metformin	500/56	Box/English	United Kingdom	Singapore	Yes/English	Unknown
28		ZOMET	500/100	Box/English	India	Singapore	None	Unknown
29	20	GLYCIPHAGE	500/60	PTP/English	India	Hong Kong	None	Unknown
30	21	Metformin	500/100	PTP/English	Unknown	Thailand	None	Unknown
31	22	Glucophage	500/100	APCFP/English	Unknown	Thailand	None	Unknown
32	23	メトホルミン塩酸塩錠500 mg (English translation: Metformin Hydrochloride 500 mg)	500/50	PTP/Japanese	Japan	Singapore	Yes/Japanese	Genuine
33	24	Metformin Hydrochloride Tablets USP	500/100	Bottle/English	India	America	Yes/English	Unknown
34	7	Metformin Hydrochloride Extended-Release Tablets, USP	500/60	Bottle/English	Canada	America	None	Unknown
35	3	GLYCIPHAGE	500/60	Bottle/English	India	India	None	Unknown
36		Metformin Hydrochloride Extended-Release Tablets, USP	1,000/60	Bottle/English	India	India	None	Unknown
37	17	Glucophage	500/60	Box/English	France	Malaysia	Yes/English	Unknown
38	24	Metformin Hydrochloride Extended-Release Table, USP	750/100	Bottle/English	India	America	Yes/English	Unknown
39		Metformin Hydrochloride Extended-Release Table, USP	500/100	Bottle/English	India	America	Yes/English	Unknown
40	15	Metsmall	500/100	Box/English	India	Singapore	None	Unknown

*PTP* press-through package, *APCFP* aluminum-plastic composite film package

There are two registered/licensed doses of metformin tablets in Japan: 250 and 500 mg. Sustained-release tablets/extended-release tablets are not approved in the country. In the United States, metformin is available at five registered/licensed doses for normal formulations (500, 625, 750, 850, and 1,000 mg) and three doses for sustained-release tablets/extended-release formulations (500, 750, and 1,000 mg).

### Visual Observations

In the visual observation of the purchased products, abnormalities such as an inappropriate description, color, stain, or scratch on the packaging or tablets were recorded. The packaging and inserts were stored as scan data, and the press-through package (PTP) sheet and aluminum-plastic composite film package (APCFP) were stored as a photograph.

### Purchase Price

We calculated the purchase price of each tablet from the Internet, excluding the shipping and import fees. The standard price for metformin prescribed under the Japanese Health Insurance System is 16.7 yen/tablet for the branded 500-mg product and 9.6–9.9 yen/tablet for generic products. The price of metformin per tablet in Japan (As of August 2, 2017) was compared with that online.

### Authenticity Investigation

A questionnaire with photographs of purchased products was sent to each manufacturer or distributor on August 14, 2017. The questionnaires contained the results of the appearance observations, authenticity tests, product names, active pharmaceutical ingredient (API), doses, forms, manufacturer name and address, batch/lot number, manufacturing license number, logo, manufacturing date, product expiration date, and a description of the outer box and instructions. We also asked whether the product was allowed to be manufactured or sold in the manufacturing country and if any countermeasures had been taken against falsified products.

### Details Given on the Website

We recorded whether the website provided the following details required by the Act on Specified Commercial Transactions [22] for personal import into Japan: name of a representative or responsible person, name of the business, address of the business, telephone number, product price, shipping fee, payment time, product delivery time, payment method, and conditions of return.

Additionally, we recorded the status of compliance with the following details required by the Act on Securing Quality, Efficacy and Safety of Products Including Pharmaceuticals and Medical Devices (Japanese Law Translation) [23]: a statement recommending consultation with a doctor or pharmacist, a description of personal importation, a statement outlining any purchase quantity limit, and a description of the medicine (e.g., name of the API, images that clearly distinguish products, or a description of the medicine’s use, dose, efficacy, and side effects), and the contact for medicine consultation.

## Materials

Metformin hydrochloride, heptanesulfonate, acetonitrile, amoxicillin trihydrate, and methanol (FUJIFILM Wako Pure Chemical Corporation, Osaka, Japan) were used for the analysis. We obtained potassium sodium chloride, phosphoric acid, potassium dihydrogen phosphate, and 0.2 N sodium hydroxide from Nacalai Tesque, Inc. (Kyoto, Japan). All other chemicals were commercially available and of analytic grade.

### Pharmacopeia Test of the Tablets

#### Quantitative Assay

Quantitative assay was conducted with reference to United States Pharmacopeia 37 (USP 37) [24] using the LC-10 AD system (Shimadzu, Kyoto, Japan) with a photodiode array detector. The column was Shim-pack CLC-ODS (M) 5 mm (4.6 mm × 25 cm, Shimadzu), and the column temperature was maintained at 30 °C using a column oven (CTO-20AC, Shimadzu). The buffer was composed of 0.5 g/L heptanesulfonate and 0.5 g/L sodium chloride in water. The pH was adjusted to 3.85 ± 0.30 with phosphoric acid. We used a mobile phase of acetonitrile: pH 3.85 buffer (1:9). The mobile phase flow rate was 0.9 mL/min, and the injection volume was 10 µL. The detection wavelength range was 232 (metformin hydrochloride tablets) or 218 nm (metformin hydrochloride sustained-release tablets). Measurements for metformin hydrochloride were performed using 10 tablets for each product. Identification of the contained components was performed by confirming that the retention time of the component matched that of the standard reagent and that the UV spectra coincided. In the first stage, the following tolerances were regarded as passing: 95.0% ≤ MEF ≤ 105.0% for normal tables [24] and 90.0 ≤ MEF ≤ 110.0% for sustained-release tablets [24]. If the samples did not reach these tolerances, they were assayed in the second stage.

#### Content Uniformity (CU) Testing

CU testing was conducted with reference to USP 37 [24]. In the first stage, a sample acceptance value (AV) ≤ 15 was regarded as passing for both normal and sustained-release tablets [24]. If the samples did not reach this tolerance, they were assayed in the second stage. If an AV of < 73.88 or > 126.88 was observed for one tablet, it was judged as a permanent fail in the first stage [24].

#### Dissolution Testing

We performed dissolution testing using a dissolution apparatus (NTR-VS6P; Toyama Sangyo Co. Ltd., Osaka, Japan) according to USP 37 [24]. Six tablets were measured per product. For normal tablets in the first stage, a tolerance of ≥ 75% of the labeled amount of metformin at a dissolution time of 45 min was regarded as passing in the dissolution test [24]. For 500-mg sustained-release tablets, the following tolerances were regarded as passing: 20%–40% for 1 h; 45%–65% for 3 h; and ≥ 80% for 10 h [24]. Additionally, the dissolution rates of 750- and 1,000-mg sustained-release tablet were determined to be appropriate as follows: 20%–40% for 1 h; 35%–55% for 2 h; 65%–85% for 6 h; and ≥ 85% for 10 h [24]. If the samples did not reach these tolerances, they were assayed in the second stage.

## Results

### Sample Collection

In total, 40 samples were obtained from 24 websites. We purchased 33 normal 500-mg tablets and 7 extended/sustained-release tablets (500 mg, five; 750 mg, one, and 1,000 mg, one). Table 1 lists the samples collected. Among the 33 normal tablets, five were branded products, and 28 were generic products. No prescription was required at any website.

### Visual Observations

Among the 500-mg tablets, one sample (No. 32) was from Singapore (Table 1), but the product PTP sheet and package insert were written in Japanese. The manufacturer listed in the package insert was “Towa Pharmaceutical Co., Ltd.” The sample had no box, and the expiration date and lot number were affixed to the package insert, which appeared to be a copy of that part of the outer box. The expiration date and lot number were not usually described in the package insert of the product sold in Japan (metformin hydrochloride tablet 500 mg MT “Towa”).

For another 500-mg sample (No. 12), the box indicated that the dose was 500 mg, but the insert stated a dose of 850 mg. From the measurement of the ingredient content, this product was considered a 500-mg tablet (Table 4).

The packaging form of the samples can be divided into four types (Table 1), namely the PTP sheet, APCFP, box (PTP sheet in a box), and bottle. Two of the PTP sheets were written in Japanese and Chinese, respectively, and all other packaging types were written in English. The package insert was enclosed for 12 of 40 samples. Among the 12 samples, the insert was written in Japanese, English, Chinese, and Thai for one, nine, one, and one product, respectively.

Among all 40 samples, only the manufacturing country was printed on a label for 29 samples and only the distributing country for one sample, whereas this information was not listed for the remaining 10 samples. The identified manufacturing countries included India, the United Kingdom, France, Thailand, Japan, and Canada, and the distribution country stated for a sample was New Zealand. Approximately 48% of samples were shipped from Singapore, and the other shipping countries were Thailand, Taiwan, the United States, India, Hong Kong, and Malaysia. One sample (No. 17) included parcel posts for both Singapore and Hong Kong (Table 1). Although the sender addresses differed, the sender was the same on both parcel posts. Because the lot numbers of this sample were identical, we defined it as a single sample.

Regarding the appearance of the tablets, sample No. 40 had a blue stain, sample No. 28 had a yellow stain, and sample No. 16 had a physical crack.

### Purchase Price

**Fig. 1:**
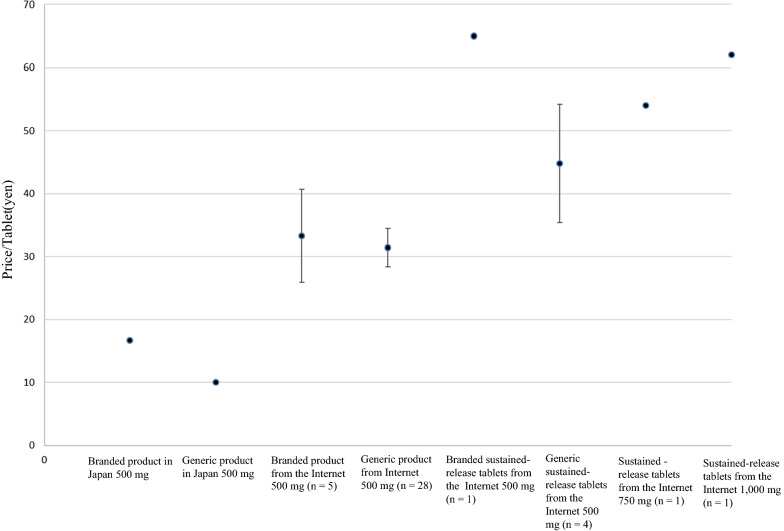
Average price per tablet (shipping fee excluded) for metformin tablets: comparison of the price of metformin tablets in Japan (branded and generic) and purchased online

Metformin is included in the National Health Insurance drug price list in Japan. Regarding price, the price of metformin 500-mg tablets was 16.7 yen/tablet for the branded product and 9.6–9.9 yen/tablet for the generic product at the time of purchase. The prices of metformin tablets purchased in this study are presented in Fig. 1. The average prices of the branded and generic 500-mg products online were (33.3 ± 7.4 (*n* = 5) and 31.4 ± 3.1 yen/tablet (*n* = 28), respectively. The online prices of the branded and generic 500-mg sustained-release tablets were 65 (*n* = 1) and 44.8 ± 9.4 yen/tablet (*n* = 4), respectively. The online prices of 750- and 1,000-mg sustained-release tablets were 54 (*n* = 1) and 62 yen/tablet (*n* = 1), respectively.

### Authenticity

A response was received from “CIPLA LTD” on August 21, 2017. We received an answer from Towa Pharmaceutical Co., Ltd. on October 24, 2017. A response was received from the “Apotex Pty Ltd” on September 8, 2017. Based on the responses, seven products were confirmed to be genuine. We sent reminders to other manufactures again in April 2018, but no response was received; thus, the authenticity of the other 33 products remains unknown.

### Evaluation of Website Details

According to the Specified Commercial Transactions Law, certain details must be provided on commercial websites (Table 2). However, only 6 of the 24 websites from which products were purchased listed all of the necessary details. We checked whether the products met the requirements of the Act on Securing Quality, Efficacy and Safety of Products Including Pharmaceuticals and Medical Devices (Table 3). Among the 24 websites, 22 did not appear to adhere fully with the recommendations for personal drug importation.

**Table 2:** Proportions of websites that met the requirements of the Specified Commercial Transactions Law

Required detail	Number of websites (%)
Name of a representative or responsible person	13 (54.0)
Name of the business	17 (71.0)
Address of the business	19 (79.0)
Telephone number	19 (79.0)
Product price	24 (100.0)
Shipping fee	21 (88.0)
Payment time	15 (63.0)
Product delivery time	22 (92.0)
Payment method	22 (92.0)
Conditions for return	21 (88.0)

*n* = 24 websites

**Table 3:** Proportions of websites that met the requirements of the Act on Securing Quality, Efficacy and Safety of Products Including Pharmaceuticals and Medical Devices

Required detail	Number of websites (%)
A statement recommending consultation with a doctor or pharmacist	7 (29.0)
A description of personal importation	17 (70.0)
A statement on the purchase quantity limit	16 (67.0)
Description of unapproved medicines or ethical medicines	24 (100.0)
The name of the product	24 (100.0)
A photograph of the product	22 (92.0)
Dose of the active ingredient	10 (41.0)
Description of the active pharmaceutical ingredient	13 (54.0)
Description of potential side effects	6 (25.0)

*n* = 24 websites

### Pharmacopeia Test of the Tablets

#### Quantification of Metformin in the Tablets

Table 4 presents the results of quantitative analysis of metformin hydrochloride samples. In quantitative analysis, 36 samples (90%) passed. The contents of sample Nos. 15, 21, and 29 (all 500-mg tablets) were 94.5 ± 1.53, 93.6 ± 5.71, and 107.0 ± 2.75%, respectively. The content of the incompatible sustained-release tablet No. 36 was 116.71 ± 16.52%, which exceeded the tolerance limit of the Pharmacopeia. [24].

**Table 4:** Results of quantity, content uniformity, and dissolution testing of metformin hydrochloride tablets

Sample No	Dose (mg)	Quantitative analysis		Content uniformity test		Dissolution test		Any fail
		Mean content (%)	Judgment	Acceptance value	Judgment	Mean dissolution rate (%)	Judgment	Judgment
1	500	95.3 ± 2.8	Pass	9.5	Pass	99.8 ± 2.5	Pass	Pass
2	500	97.5 ± 3.2	Pass	13.7	Pass	95.0 ± 1.7	Pass	Pass
3	500	101.2 ± 2.4	Pass	5.9	Pass	99.2 ± 1.0	Pass	Pass
4	500	99.1 ± 3.5	Pass	8.4	Pass	102.0 ± 1.9	Pass	Pass
5	500	97.3 ± 1.8	Pass	5.6	Pass	96.4 ± 3.6	Pass	Pass
6	500	95.5 ± 1.3	Pass	6.1	Pass	96.6 ± 1.4	Pass	Pass
7	500	96.5 ± 2.3	Pass	7.6	Pass	96.1 ± 1.2	Pass	Pass
8	500	97.5 ± 5.2	Pass	13.7	Pass	95.5 ± 2.4	Pass	Pass
9	500	96.7 ± 4.5	Pass	12.6	Pass	104.2 ± 2.1	Pass	Pass
10	500	97.3 ± 1.0	Pass	3.6	Pass	95.0 ± 2.7	Pass	Pass
11	500	101.7 ± 1.5	Pass	3.6	Pass	102.2 ± 2.1	Pass	Pass
12	500	96.2 ± 1.2	Pass	2.8	Pass	100.0 ± 3.6	Pass	Pass
13	500	95.8 ± 2.8	Pass	9.6	Pass	99.9 ± 1.8	Pass	Pass
14	500	95.6 ± 2.1	Pass	7.9	Pass	95.4 ± 2.6	Pass	Pass
15	500	94.5 ± 1.5	Fail	7.7	Pass	98.4 ± 1.9	Pass	Fail
16	500	103.9 ± 1.4	Pass	5.7	Pass	99.5 ± 3.1	Pass	Pass
17	500	99.5 ± 3.7	Pass	8.9	Pass	100.4 ± 3.3	Pass	Pass
18	500	97.1 ± 1.2	Pass	4.2	Pass	106.7 ± 11.1	Pass	Pass
19	500	96.4 ± 1.9	Pass	6.7	Pass	98.7 ± 2.0	Pass	Pass
20	500	99.5 ± 0.7	Pass	1.7	Pass	99.8 ± 0.9	Pass	Pass
21	500	93.6 ± 5.7	Fail	18.6	Fail	102.2 ± 1.8	Pass	Fail
22	500	101.7 ± 2.9	Pass	7.0	Pass	103.8 ± 2.2	Pass	Pass
23	500	96.3 ± 1.5	Pass	5.7	Pass	105.5 ± 4.8	Pass	Pass
24	500	98.5 ± 0.7	Pass	1.7	Pass	98.1 ± 0.9	Pass	Pass
25	500	96.3 ± 3.8	Pass	11.3	Pass	102.0 ± 1.7	Pass	Pass
26	500	96.5 ± 1.4	Pass	5.5	Pass	99.6 ± 4.0	Pass	Pass
27	500	96.2 ± 4.2	Pass	12.4	Pass	99.5 ± 3.2	Pass	Pass
28	500	95.9 ± 1.8	Pass	7.1	Pass	99.0 ± 3.3	Pass	Pass
29	500	107.0 ± 2.8	Fail	12.1	Pass	101.6 ± 1.1	Pass	Fail
30	500	99.1 ± 1.8	Pass	4.2	Pass	104.7 ± 1.5	Pass	Pass
31	500	99.0 ± 1.9	Pass	4.6	Pass	103.4 ± 1.2	Pass	Pass
32	500	97.8 ± 1.1	Pass	3.3	Pass	100.9 ± 5.0	Pass	Pass
33	500	96.1 ± 1.4	Pass	5.8	Pass	100.0 ± 1.6	Pass	Pass
34	500	96.5 ± 4.7	Pass	13.3	Pass	1 h: 33.7 ± 4.7 3 h: 60.0 ± 1.8 10 h: 95.6 ± 4.0	Pass	Pass
35	500	104.3 ± 5.8	Pass	16.6	Fail	1 h: 32.8 ± 0.4 3 h: 60.8 ± 1.6 10 h: 99.8 ± 4.8	Pass	Fail
36	1,000	116.71 ± 16.5	Fail	54.9	Fail	1 h:33.0 ± 1.6 2 h:49.3 ± 1.2 6 h: 87.0 ± 6.3 10 h: 96.7 ± 3.9	Fail	Fail
37	500	109 ± 3.0	Pass	14.8	Pass	1 h: 29.1 ± 1.5 3 h: 57.0 ± 10.3 10 h: 93.2 ± 6.6	Pass	Pass
38	750	100.5 ± 8.7	Pass	21.1	Fail	1 h: 30.5 ± 1.4 2 h: 46.2 ± 5.3 6 h: 83.4 ± 4.1 10 h: 94.7 ± 2.2	Pass	Fail
39	500	104.3 ± 2.8	Pass	9.6	Pass	1 h: 35.8 ± 4.8 3 h:63.9 ± 5.9 10 h: 98.6 ± 2.8	Pass	Pass
40	500	99.2 ± 7.5	Pass	23.0	Fail	1 h: 38.7 ± 1.4 3 h: 68.6 ± 3.3 10 h: 97.6 ± 2.2	Fail	Fail

#### CU Testing

In total, 35 samples (87.5%) passed CU testing (Table 4). Among the failing samples, AV ranged 16.6–54.9.

#### Dissolution Testing

Table 4 presents the results of dissolution testing for the samples. In total, 38 samples (95%) passed. Two samples that failed testing were sustained-release/extended-release tablets. The dissolution rate of tablet No. 40 (500-mg sustained-release tablet) was 68.6 ± 3.25% at 3 h, which exceeded the tolerance limit. The dissolution rate of tablet No. 36 (1,000-mg extended-release tablet) was 87.03 ± 6.28% at 6 h, which also exceeded the tolerance limit.

## Discussion

In this study, we found that 4 of 24 websites (17%) for personal import agencies advertised the API, efficacy, and names of products (metformin hydrochloride sustained-release tablet) not approved in Japan, although article 68 of the Act on Securing Quality, Efficacy and Safety of Products Including Pharmaceuticals and Medical Devices prohibits advertising of unregistered/unlicensed medicines, and advertisements related to the name, manufacturing method, efficacy, effect, or performance of unregistered/unlicensed medicine are also prohibited [23].

Additionally, it has become clear that unregistered/unlicensed medicines can be obtained without a prescription. Unregistered/unlicensed medicines available online have not been evaluated for safety in Japan, and thus, they are not eligible for the medicine side effects relief system [25]. Personally imported drugs carry certain health risks. Additionally, the Ministry of Health, Labour and Welfare of Japan has been providing information and warnings about the personal import of medicines to patients and consumers, as well as monitoring advertisements for medicine promotion on the Internet [25, 26]. For unregistered/unlicensed medicines, the possibility of compassionate use cannot be excluded. However, the use of unregistered/unlicensed medicines should be avoided for general consumers. The unrecognized/unlicensed drugs can be obtained in Japan via the Internet; this situation may exist in other countries as well. To prevent adverse outcomes caused by personally imported drugs, it is necessary to raise awareness to ensure that consumers do not carelessly import pharmaceutical products.

We obtained one Japanese product (No. 32) among the products examined in this study (Table 1). This is one of the problems of the personal import of medicines in Japan. Medicines manufactured in Japan will be delivered from the manufacturing company to hospitals and pharmacies through domestic wholesalers and ultimately delivered to consumers. However, the availability of a Japanese product online suggested that an unauthorized distribution channel of drugs exists. This channel differed from the original distribution channels of imported agents. Because of the existence of unauthorized distribution channels, the quality of the medicine can be altered because of improper transportation and storage even if the product was manufactured in Japan.

Although metformin is a prescription medicine in Japan, online wholesalers do not confirm prescriptions. Concerning the outer packaging (Table 1), six box-packaged samples from Singapore and one sample from India in blister packaging were deemed genuine. However, because of insufficient information from the manufacturer or distributor on the package and the low response rate for the authenticity survey, we cannot identify and summarize the rules and basis for judging the authenticity or quality of the products. It was difficult to determine whether the labeled manufacturing or distributing country on the package was accurate because the authenticity of samples was not determined.

Visual observation confirmed that several samples had physical cracks or stains. Although all of these samples passed quality testing, the cracks or stains may have been caused by lower-quality excipients or improper manufacturing practices, or may have been introduced during transportation because of defective packaging. These deficiencies might affect the health of consumers.

Consumers who obtain medicines via personal import agencies without guidance from a doctor or pharmacist possibly refer to the package and instructions of the product when using the drugs. However, the packaging and insert for 98% of the purchased samples were not written in Japanese, and inserts and packaging materials written in foreign languages do not provide sufficient information to patients. Additionally, the dose described in the insert of one sample did not match the actual dose. Inappropriate drug use may result in side effects, which can threaten patients’ health.

The online price of one 500-mg metformin tablet was higher than that from a regular Japanese medical institution (Fig. 1). Therefore, there are no cost advantages to purchasing drugs online. None of the websites required a prescription to purchase metformin and unauthorized/unlicensed doses could be obtained. This encourages the import of drugs that can cause health effects associated with improper use.

In total, four and five samples failed quantitative analysis and CU testing, respectively (Table 4). If the content of the active ingredient is insufficient or inconsistent, therapeutic efficacy cannot be achieved. Long-term continuous use of ineffective drugs may increase the risks of complications such as retinopathy, nephropathy, neurological disorders, and heart disease.

For the dissolution test, two samples (both extended-release/sustained-release tablets) failed specifications (Table 4). The sustained-release system of two samples was broken, and appropriate sustained release was not observed. However, because the result only slightly deviated from the pharmacopeia standard, the products are not expected to cause a rapid increase in blood glucose concentrations, which increases the risks of adverse outcomes.

One limitation of the study was that we only tested 40 samples and evaluated 24 websites. The research object was only one drug, and the results do not reflect the quality of all medicines available online. Expanding the number of samples may provide more comprehensive information and permit more relevant discoveries. Another limitation was that most manufacturers did not complete the authenticity survey, and thus, we could not verify the authenticity of most products. However, the circulation of low-quality medicines on the Internet was confirmed. This study indicates the need for consumers to avoid purchasing medicines on the Internet. Additionally, the findings also revealed that active cooperation between manufacturers and regulatory authorities is necessary for improving the quality of medicines in circulation.

## Conclusions

This study clarified that metformin tablets of poor-quantity and unregistered/unlicensed doses can be personally imported into Japan without a prescription via the Internet. It is difficult to judge the quality of medicines based on the outer packaging. Because medicines can easily be imported via the Internet, consumers must be aware of the existence of falsified or poor-quantity medicines and the dangers of personal import. Additionally, relevant regulatory authorities should provide consumers with information and warnings to increase awareness of this issue. Moreover, it may be necessary to monitor or block illegal personal import agencies or senders to ensure the safety of consumers.
